# A Siderophore-Encoding Plasmid Encodes High-Level Virulence in Escherichia coli

**DOI:** 10.1128/spectrum.02528-21

**Published:** 2022-05-23

**Authors:** Han Wang, Qi Xu, Kaichao Chen, Bill Kwan Wai Chan, Lianwei Ye, Xuemei Yang, Miaomiao Xie, Xiaobo Liu, Hongyuhang Ni, Edward Wai Chi Chan, Sheng Chen

**Affiliations:** a Department of Infectious Diseases and Public Health, Jockey Club College of Veterinary Medicine and Life Sciences, City University of Hong Kong, Kowloon, Hong Kong; b National Engineering Laboratory for Deep Process of Rice and By-Products, Hunan Key Laboratory of Grain-Oil Deep Process and Quality Control, Hunan Key Laboratory of Processed Food for Special Medical Purpose, College of Food Science and Engineering, Central South University of Forestry and Technology, Changsha, Hunan, China; c State Key Lab of Chemical Biology and Drug Discovery, Department of Applied Biology and Chemical Technology, The Hong Kong Polytechnic University, Hung Hom, Kowloon, Hong Kong; University of Hong Kong

**Keywords:** conjugation, virulence plasmid, avian pathogenic *E. coli*, hypervirulent *K. pneumoniae*, transmission

## Abstract

A plasmid that harbored the virulence factors highly like those of the virulence plasmid commonly found in clinical hypervirulent Klebsiella pneumoniae strains was detected in a foodborne Escherichia coli strain EC1108 and designated p1108-IncFIB. This virulent-like plasmid was found to be common in E. coli from various sources. To understand the contribution of this plasmid to the virulence of E. coli, plasmid p1108-IncFIB in strain EC1108 was first cured to generate strain EC1108-PC. The virulence plasmid p15WZ-82_Vir in Klebsiella pneumoniae strain 15WZ-82 was then transmitted to EC1108-PC to produce the transconjugant, EC1108-PC-TC to assess the contribution of this virulence plasmid to the virulence level of E. coli. During the process of conjugation, p15WZ-82_Vir was found to be evolved into p15WZ-82_int, which underwent homologous recombination with a plasmid encoding a carbapenemase gene, *bla*_NDM-1_, p1108-NDM, in EC1108-PC. Comparison between the level of virulence in the EC1108, EC1108-PC-TC, and EC1108-PC through serum and macrophage resistance assay, as well as animal experiments, confirmed that plasmid p1108-IncFIB encoded a high level of virulence in E. coli, yet the fusion plasmid derived from p15WZ-82_Vir did not encode virulence but instead imposed a high fitness cost in the E. coli strain EC1108-PC-TC. These findings indicate that E. coli strains carrying the virulence plasmid p1108-IncFIB in multidrug-resistant (MDR) strains may also impose serious public health threats like that of hypervirulent Klebsiella pneumoniae strains harboring the p15WZ-82_Vir plasmid.

**IMPORTANCE** Acquisition of pLVPK-like virulence plasmid by Klebsiella pneumoniae converts it to hypervirulent K. pneumoniae (HvKP), which has become one of the most important clinical bacterial pathogens. The potential of transmission of this virulence plasmid and its contribution to the virulence of other Enterobacteriaceae, such as E. coli, are not clear yet. In this study, we showed that pLVPK-like virulence plasmid exhibited fitness costs and did not contribute to the virulence in E. coli. However, we identified a novel virulence plasmid, p1108-IncFIB, that encodes similar siderophore genes as those of pLVPK from a foodborne E. coli strain and showed that p1108-IncFIB encoded a high level of virulence in E. coli. BLAST of E. coli genomes from GenBank showed that these siderophore genes were widespread in clinical E. coli strains. Further studies are warranted to understand the impact of this plasmid in the control of clinical infections caused by E. coli.

## INTRODUCTION

Klebsiella pneumoniae is a common Gram-negative bacillus that causes a variety of human clinical infections, especially in immunocompromised patients (1). Hypervirulent K. pneumoniae (HvKP) is a continually evolving pathotype that encodes a significantly higher level of virulence than the classical Klebsiella pneumoniae (cKP) strains and may cause infections in healthy individuals (2). The evolution of cKP to HvKP is through the acquisition of a pLVPK-like virulence plasmid, which contains several virulence genes, including those encoding salmochelin (*iroBCDN*), and aerobactin (*iucABCD-iutA*), a regulator of mucoid phenotype (*rmpA*), and a regulator of mucoid phenotype 2 (*rmpA2*) (3). Recent studies showed that this virulence plasmid could be transferred to both K. pneumoniae and Escherichia coli through various mechanisms (3, 4). However, it is not clear whether this virulence plasmid could also mediate the enhancement of virulence in E. coli.

We recently recovered a virulence plasmid from an E. coli strain isolated from a chicken meat sample. This plasmid is structurally similar to p1ColV5155 (accession number: CP005931), which is carried by an avian pathogenic Escherichia coli (APEC) strain IMT5155 isolated from a chicken sample in Germany (5). Exhibiting the potential to cause typical clinical symptoms of avian colibacillosis, this plasmid also harbored a DNA region that encodes the salmochelin siderophore system (*iroBCDEN*), as well as the aerobactin cluster (*iucABCD*-*iutA*). Owing to the functional role of the siderophore system in mediating high virulence in K. pneumoniae, it is necessary to investigate the degree of contribution of this plasmid to virulence expression in E. coli. Avian pathogenic Escherichia coli (APEC) is an important extraintestinal Escherichia coli (ExPEC) that causes avian colibacillosis (6). Several studies showed that the genomic features of APEC and human ExPEC are highly similar, with many genetic regions being homologous to each other. They also contain common virulence-related genes, cause similar disease patterns, and have similar evolutionary backgrounds (7, 8). Therefore, we speculate that some APEC strains may pose a severe threat to public health (9). In this study, we investigated whether the pLVPK-like and p1ColV5155-like virulence plasmids encode high-level virulence in E. coli.

## RESULTS

### Comparison of genetic makeups of virulence plasmids recovered from E. coli and K. pneumoniae.

**FIG 1:**
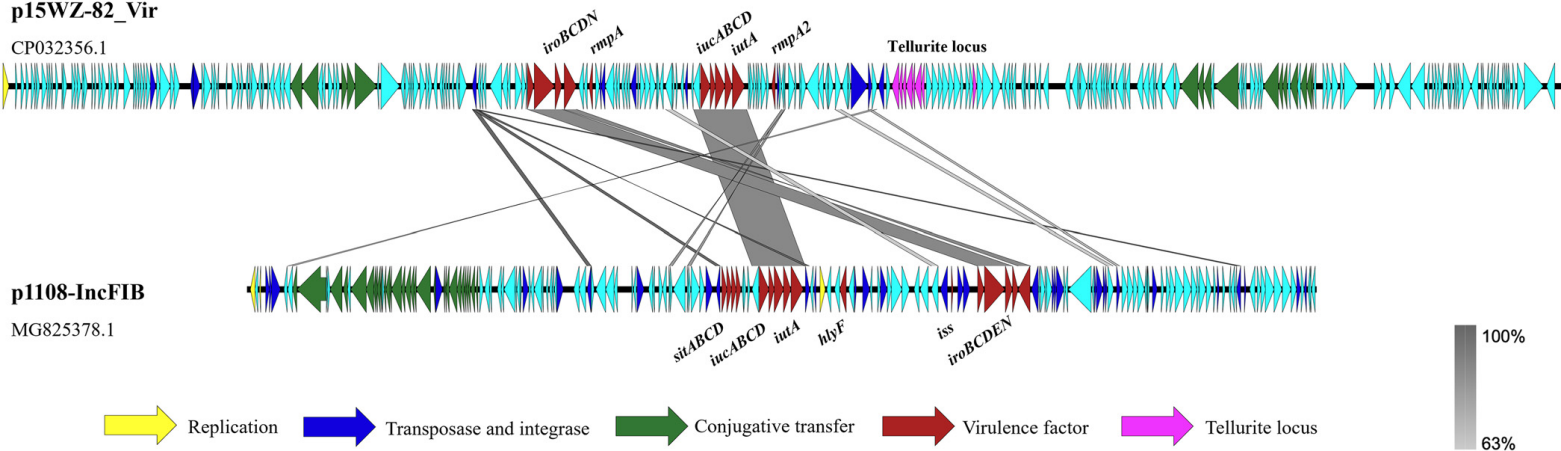
Alignment of the virulence plasmid p1108-IncFIB recovered from strain EC1108 with the virulence plasmid p15WZ-82_Vir harbored by strain 15WZ-82 by EasyFig. Yellow, the gene encoding replication function; dark blue, genes encoding integrase and transposase; green, genes encoding the conjugative transfer protein (*tra*, *trb*); red, genes encoding virulence factors; pink, genes of the tellurite locus. The shade denotes regions of genetic similarity between the two plasmids; the scale in the right corner reveals the degree of similarity.

The whole-genome sequence of Escherichia coli strain EC1108 was obtained. Bioinformatics analysis showed that it belonged to an avian pathogenic Escherichia coli (APEC) strain with a serotype of O-non-type (ONT): H28 and sequence type (ST) type of E. coli 359. It shared the same serotype, ST type, and similar virulence factor with human extraintestinal pathogenic Escherichia coli (ExPEC) strains that are known to cause zoonotic diseases. The complete sequence of a plasmid that encodes various siderophores was obtained and designated p1108-IncFIB. Another virulence plasmid tested in this study was recovered from a hypervirulent K. pneumoniae (HvKP) strain 15WZ-82 and designated p15WZ-82_Vir, which was reported in our previous study (3). Sequence alignment of these two plasmids showed they shared approximately 22.9 kb of identical sequences, including the salmochelin (*iroBCDN*) and aerobactin (*iucABCD-iutA*)-encoding genes (99% similarity). In addition, p1108-IncFIB also carried genes encoding the iron/manganese ABC transporter (*sitABCD*), hemolysin (*hlyF*), increased serum survival factor (*iss*), and salmochelin (*iroE*), whereas plasmid p15WZ-82_vir was found to carry the gene encoding the regulator of the mucoid phenotype (*rmpA*) and the regulator of mucoid phenotype 2 (*rmpA2*) (10). Each of the above virulence genes was flanked by transposable elements (Fig. 1).

### Distribution of virulence plasmids in E. coli.

**FIG 2:**
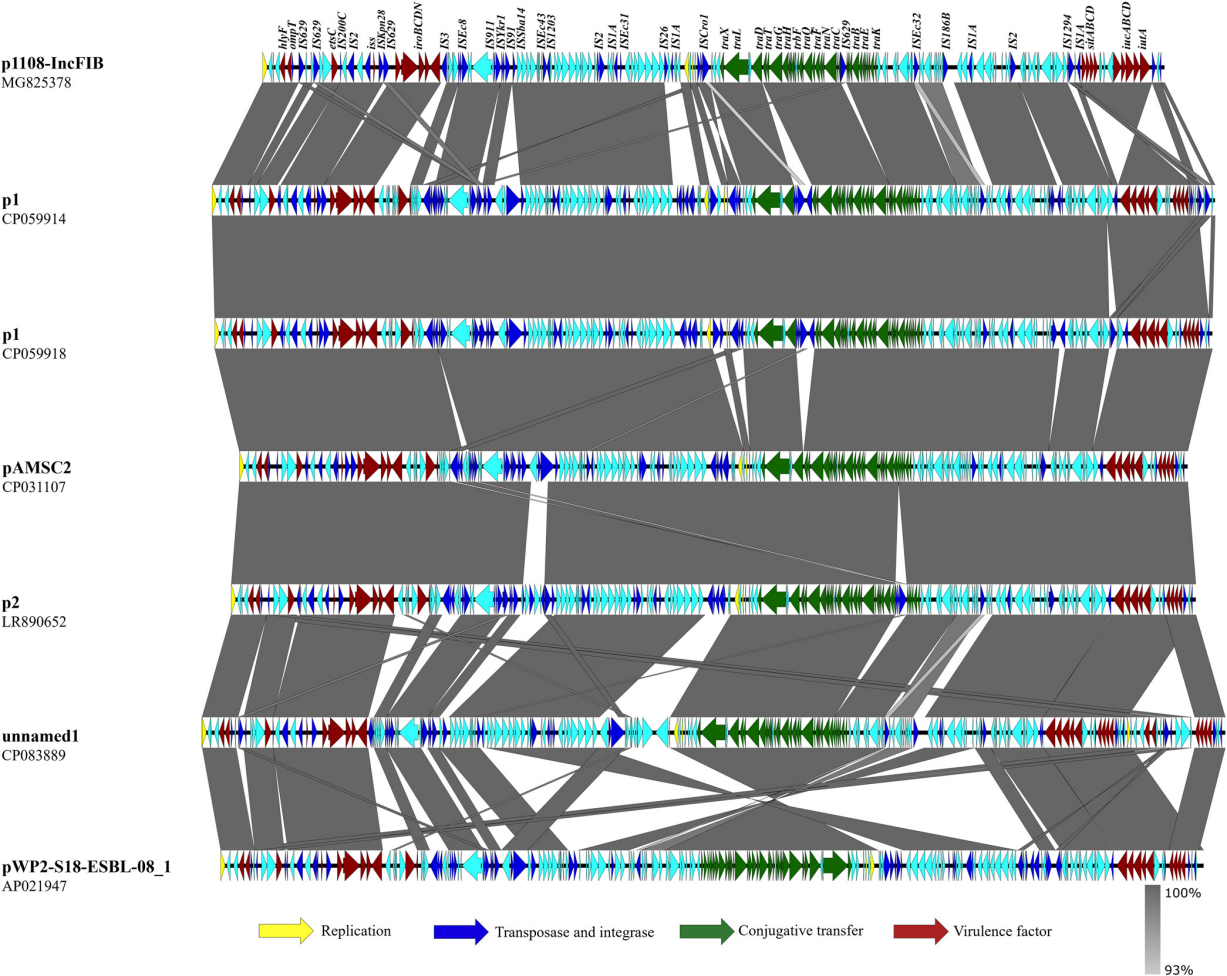
Genetic alignment of the virulence plasmid p1108-IncFIB with other virulence plasmids from E. coli deposited in NCBI database using Easyfig. Liner alignment of plasmid p1108-IncFIB (MG825378), p1 (accession number: CP059914), p1 (accession number: CP059918), pAMSC2 (accession number: CP031107), p2 (accession number: LR890652), unnamed1 (accession number: CP083889) and pWP2-S18-ESBL-08_1 (accession number: AP021947). Yellow, gene encoding replication initiation protein; blue, insertion sequence; red, virulence gene; green, gene encoding plasmid conjugative transfer protein, Tra.

Whole-genome sequencing (WGS) analysis demonstrated that plasmid p1108-IncFIB was 193,873 bp (bp) in size, contained the IncFIB replicon, and comprised 209 predicted coding sequences (CDs), with a CG content of 50. 0%. Two clusters of virulence determinants, namely, *iroBCDEN* and *iucABCD-sitABCD*, were found to be surrounded by the insertion sequences IS*629* and IS*3*/*IS*2, respectively. BLASTn analysis showed that p1108-IncFIB-like plasmids were rare in the NCBI database. Only six plasmids were found to exhibit high homology (99.76% to 99.84% identify, 95% to 96% coverage) to this plasmid, including p1 (accession number: CP059918), p2 (accession number: LR890652), pAMSC2 (accession number: CP031107), p1 (accession number: CP059914), pWP2-S18-ESBL-08_1 (accession number: AP021947) and unnamed1 (accession number: CP083889), which can be found in an extensive range of bacterial hosts and are detectable in turkey, panda dung, wastewater treatment plant effluent and blood samples of patients from Canada, China, Japan, and the USA, respectively (Fig. 2). Among these plasmids, antimicrobial resistance genes (AMRs) cannot be detected, whereas many IS sequences were observed. Insertion or deletion events involving these insertion elements were the key mechanism underlying the divergence of these plasmids. Besides, p1108-IncFIB-like plasmids in the NCBI database have the extra virulence-associated gene *cvaC* and the backbone region of *tra* genes responsible for plasmid conjugation, which were extremely conserved, indicating that this kind of virulence plasmid may pose a potential threat to both animals and humans because of their high transmissibility (Fig. 2).

To screen for the prevalence of these virulence factors in other bacterial strains, we randomly selected 2915 genome sequences collected from 4 main categories, including animal (poultry and swine), meat (pork chop and chicken), human clinical samples (blood, urine, sputum, and feces) and environmental samples deposited in NCBI database to screen for *iroBCDEN*, *iucABCDiutA* and *sitABCD* genetic loci in these strains. Our data showed that the presence of these three genetic loci in E. coli strains from animals was about 26.1%, 34.0%, and 39.1, respectively, with prevalence in poultry higher than in swine; the prevalence was about 22.76%, 28.21%, and 34.85%, respectively, in meat with higher prevalence in chicken; the prevalence in clinical isolates was 11.28%, 48.83%, and 57.02%, respectively. The prevalence in environmental isolates was 13.03%, 16.94%, and 25.08, respectively (Table 1). Some proportion of the strains also carried all these three genetic loci (Table 1). These data suggested that the prevalence of these three genetic loci in E. coli strains is quite high, particularly in clinical E. coli isolates, which implied the potentially important role of these genetic loci on the pathogenesis of E. coli and warranted further investigation.

**TABLE 1:** Distribution of three toxin cluster genes among deposited E. coli strains collected from clinical, environmental, animal, and meat samples in the NCBI database

Category	Source	No. of samples	No. of strains containing			
			*iroBCDEN*	*iucABCDiutA*	*sitABCD*	Three cluster
Animal	Swine	584	65 (11.13%)	131 (22.43%)	157 (26.88%)	54 (9.25%)
	Poultry	447	205 (45.86%)	220 (49.22%)	246 (55.03%)	151 (33.78%)
	Total	1031	270 (26.1%)	351 (34.0%)	403 (39.1%)	205 (19.8%)
						
Meat	Pork chop	317	56 (17.67%)	55 (17.35%)	81 (25.55%)	45 (14.20%)
	Chicken	320	89 (27.81%)	124 (38.75%)	141 (44.06%)	57 (17.81%)
	Total	637	145 (22.76%)	179 (28.21%)	222 (34.85%)	102 (16.01%)
						
Clinical (human)	Feaces	611	45 (7.36%)	288 (47.14%)	309 (50.57%)	30 (4.91%)
	Urine	183	37 (20.22%)	109 (59.56%)	144 (78.69%)	28 (15.3%)
	Sputum	77	14 (18.18%)	29 (37.66%)	38 (49.35)	13 (16.88%)
	Blood	69	10 (14.49%)	33 (47.83%)	45 (65.22%)	4 (5.80%)
	Total	940	106 (11.28%)	459 (48.83%)	536 (57.02%)	75 (7.98%)
						
Environment		307	40 (13.03%)	52 (16.94%)	77 (25.08%)	22 (7.17%)

### Creation of E. coli 1108 with and without carrying virulence plasmids.

To investigate the degree of contribution of these virulence plasmids to virulence expression, we created E. coli 1108 strains with and without harboring the virulence plasmid. First, plasmid curing was performed, and a virulence plasmid-cured strain designated EC1108-PC was successfully obtained. PCR targeting the siderophore genes was then performed with results showing that EC1108-PC did not harbor the *iucC* gene located in the virulence plasmid. In addition, S1-PFGE was also performed on EC1108-PC and confirmed that this strain did not carry a plasmid with a size of ~190kb. On the other hand, XbaI PFGE confirmed that EC1108-PC exhibited an identical PFGE profile as EC1108, suggesting that this strain (EC1108-PC) was EC1108 without the virulence plasmid.

**FIG 3:**
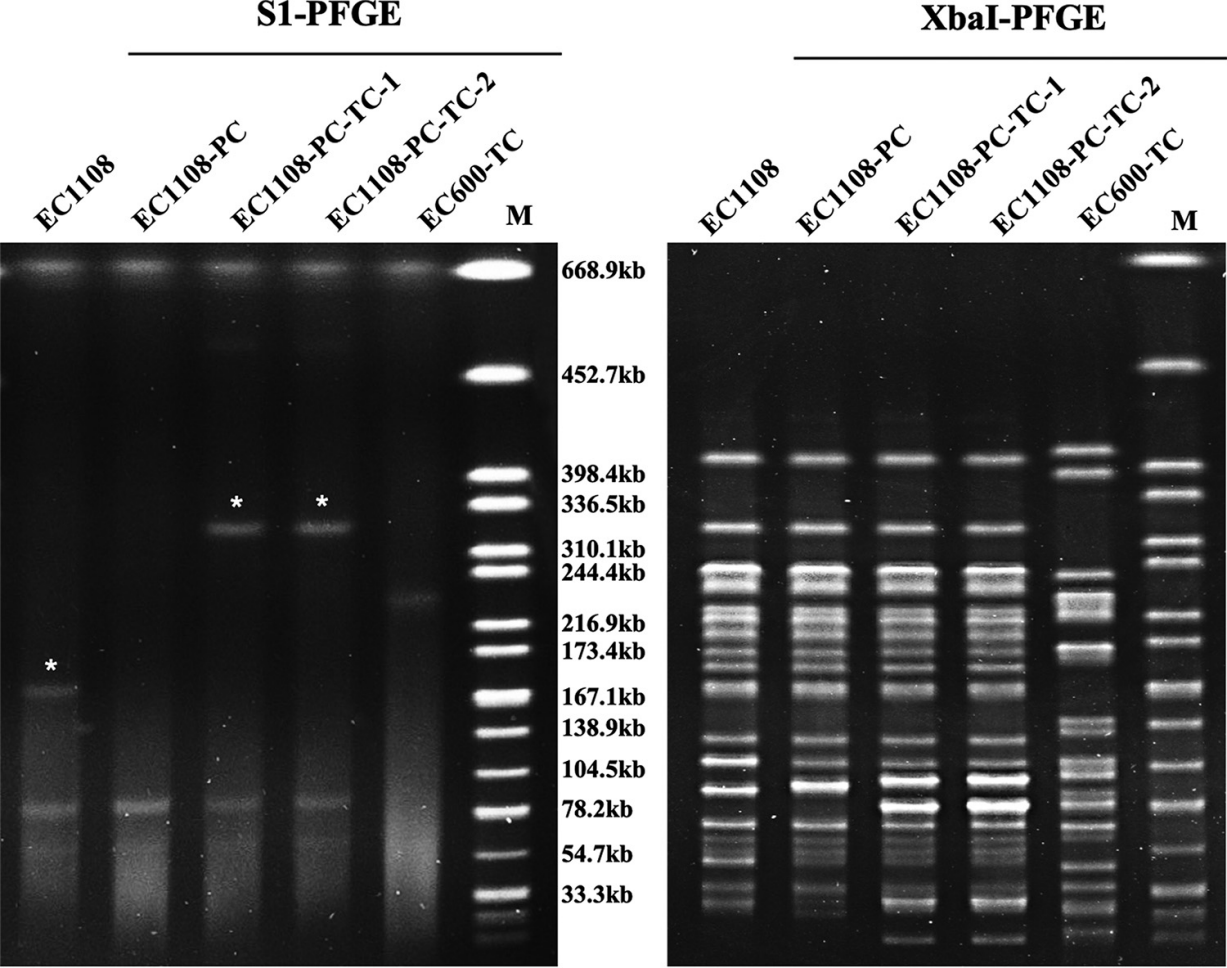
S1-PFGE and XbaI PFGE of EC1108, the recipient strain EC1108-PC with the virulence plasmid cured, the donor strain EC600-TC, and their transconjugant, EC1108-PC-TC. Asterisk in lane EC1108 indicated virulence plasmid from EC1108, p1108-IncFIB; Asterisks in EC1108-PC-TCs indicated the hybrid plasmid, p1108-NDM_Vir in EC1108, which was formed by K. pneumoniae virulence plasmid, p15WZ-82_Vir and *bla*_NDM_-bearing plasmid from EC1108, p1108-NDM. EC600-TC indicated the E. coli EC600 strain carrying K. pneumoniae virulence plasmid, p15WZ-82_Vir, which was shown in the lane of EC600-TC. M represents the XbaI PFGE of H9812 that was used as the marker.

To check whether the virulence plasmid from HvKP encodes virulence in E. coli, we conducted a conjugation experiment to transfer the virulence plasmid to EC1108-PC. Our previous studies showed that virulence plasmid from CR-HvKP strain, p15WZ-82_Vir, could be directly conjugated to Escherichia coli EC600 to generate EC600-TC, which then exhibited higher conjugation efficiency (10^−2^ to 10^−4^) as a donor strain than K. pneumoniae 15WZ-82 (10^−6^ to 10^−8^) (3). Therefore, EC600-TC was selected as the donor of p15WZ-82_Vir in this study. The XbaI-PFGE profiles of EC1108, EC1108-PC, and EC1108-PC-TC were almost identical, indicating that these three E. coli strains were closely related. S1-PFGE showed that EC1108-PC lacked a plasmid of a size of 194 kb compared with EC1108, indicating that the virulence plasmid, p1108-IncFIB, was cured. Interestingly, there was only one plasmid of 282 kb in the donor strain (EC600-TC), while an extra plasmid with a size of about 380 kb could be recovered in the transconjugants (EC1108-PC-TC), suggesting that the virulence plasmid (p15WZ-82_Vir) might undergo genetic recombination with other DNA fragments in EC1108 (Fig. 3). MICs were determined for the transconjugants, with results showing that it exhibited an identical antimicrobial resistance (AMR) profile as EC1108, plus additional phenotypic resistance to potassium tellurite resistance, suggesting that the virulence plasmid has been transferred to EC1108 (Table 2). PCR assay targeting *rmpA2* also confirmed that the transconjugants have obtained the virulence plasmid (11). The conjugation efficiency from the fusion plasmid carrying p15WZ-82_Vir to EC1108-PC was determined to be around 10^−5^ to 10^−6^.

**TABLE 2:** Phenotypic and genetic characteristics of four E. coli strains tested in this study

Strain ID	Bacteria Species	MIC (μg/mL)											
		AMP	AMK	CAZ	CIP	CLO	CTX	GEN	KAN	TET	TE	*iucC*	*rmpA2*
EC600-TC^*a*^	E. coli	4	4	2	<1	4	<1	<1	4	2	>64	+^*b*^	−
EC1108-PC	E. coli	>64	>64	>64	>64	>64	>64	>64	>64	>64	<1	−	−
EC1108-PC-TC	E. coli	>64	>64	>64	>64	>64	>64	>64	>64	>64	>64	−	+
15WZ-82	K. pneumoniae	2	<1	<1	<1	4	<1	<1	2	<1	>64	−	+

a E. coli strain EC600 carrying the conjugative virulence plasmid, p15WZ-82_Vir, was used as a donor strain in conjugation studies.

b ‘+’, positive for *iucC* and *rmpA2*; ‘−’, negative for *iucC* and *rmpA2*.

### Genetic characteristics of the conjugative fusion plasmid.

Complete sequences of plasmids in transconjugants were obtained by using the long-read Oxford Nanopore Technologies MinION platform (Nanopore, Oxford, United Kingdom) and the Illumina NextSeq 500 platform (Illumina, San Diego, CA). The complete plasmid sequence of EC1108 has been determined in another unpublished study, which showed that this E. coli strain carried seven different plasmids, including p1108-Col (2096 bp), p1108-MCR (64906 bp), p1108-IncY (96082 bp), p1108-NDM (96688 bp), p1108-CMY2 (98157 bp), p1108-ermB (101660 bp) and p1108-IncFIB (193,873bp). EC1108-PC harbored all other six plasmids in EC1108 except p1108-IncFIB. A total of six plasmids were identified in the transconjugant, including p1108-Col, p1108-MCR, p1108-IncY, p1108-CMY2, p1108-ermB as well as a plasmid with the size of ~380kb. Comparative analysis of plasmids from EC1108 and the transconjugant showed that the virulence plasmid p15WZ-82_Vir was fused to another plasmid (p1108-NDM) carrying a *bla*_NDM-1_ gene and became a conjugative fusion plasmid in EC1108-PC-TC, which is consistent with the S1-PFGE result reported above (Fig. 3).

**FIG 4:**
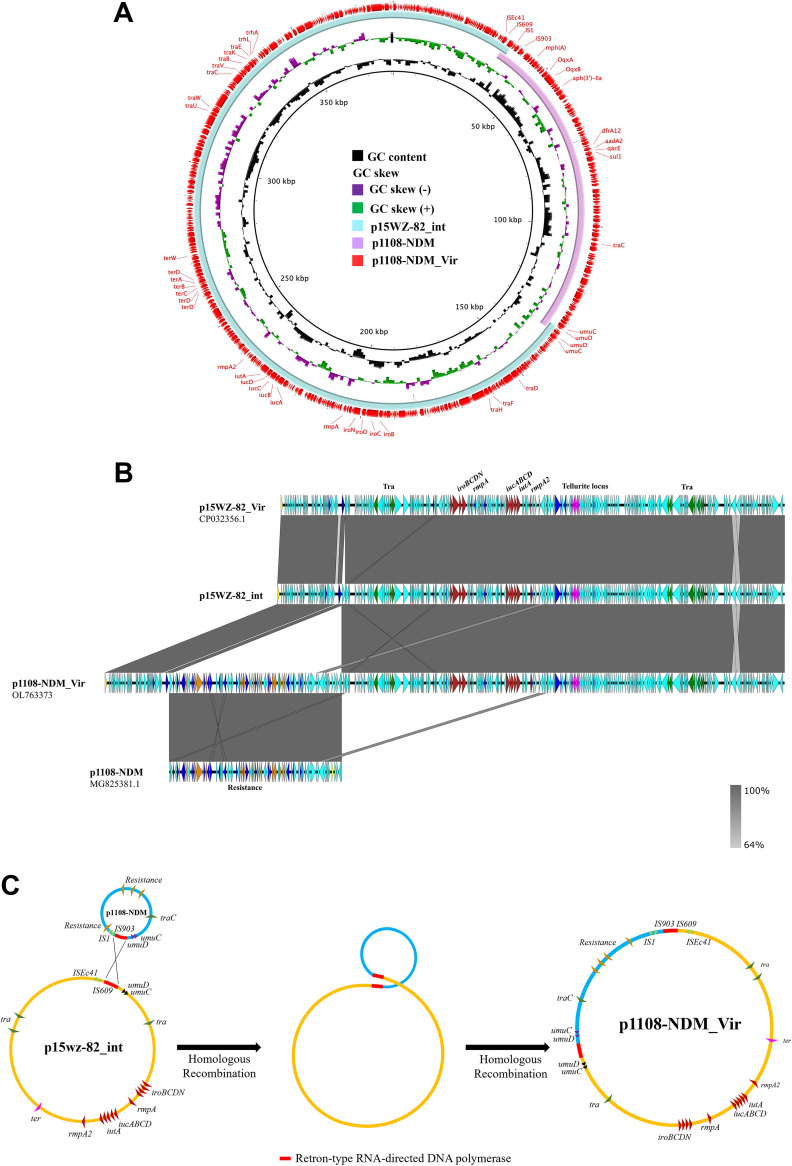
Recombination of p15WZ-82_Vir and *bla*_NDM_-bearing plasmid, p1108-NDM to form the fusion plasmid p1108-NDM_Vir during conjugation. (A) Alignment of p1108-NDM_Vir with p1108-NDM, p15WZ-82_Vir, and p15WZ-82_int by BRIG. (B) Alignment of p1108-NDM_Vir with p1108-NDM, p15WZ-82_Vir, and p15WZ-82_int by easyfig. (C) Mechanism of fusion plasmid, in which p1108-NDM_Vir forms through homologous recombination.

Plasmid analysis showed that the fusion plasmid contained an extra 1934 bp sequence that encodes a Retron-type reverse RNA-directed DNA polymerase, compared to the two plasmids mentioned above. In the intermediate plasmid p15WZ-82-int, the upstream region of Retron-type reverse transcriptase contained the *ISEc41*/*IS609* family transposase-encoding elements, and the downstream region contained two error-prone repair genes, *umuD*/*umuC*. Also, in p1108-NDM, the upstream and downstream regions harbor the *umuC*/umuD and IS*1*/IS*903* family transposase-encoding elements, respectively. It was noteworthy that the 1934 bp sequence appeared twice in the fusion plasmid, indicating that it was the homologous region (HR) involved in the recombination of the two plasmids. These two homologous regions were identical and shared 99% homology. A similar recombination event involving p15WZ-82_Vir during conjugation was reported by Yang et al. (12). Based on the genetic features of the plasmids analyzed in this work, we proposed the plasmid recombination process as follows. The plasmid p1108-NDM was initially inserted into the 1934 bp region in p15WZ-82_Vir and generated the intermediate plasmid p15WZ-82_int. A crossover homologous recombination event then occurred in the homologous region, resulting in the integration of p15WZ-82_Vir with p1108-NDM and the formation of a conjugative fusion plasmid, p1108-NDM_Vir, which contained both virulence and resistance genes (Fig. 4). However, we could not detect the 284,205 bp of p15WZ-82_int through S1-PFGE because the intermediate plasmid was only transient in strain EC1108-PC-TC. Upon culturing the bacteria for a long time, only the fusion plasmid p1108-NDM_Vir could be retained, while p15WZ-82-int was lost.

### EC1108 exhibits strong resistance to serum and macrophage killing.

**FIG 5:**
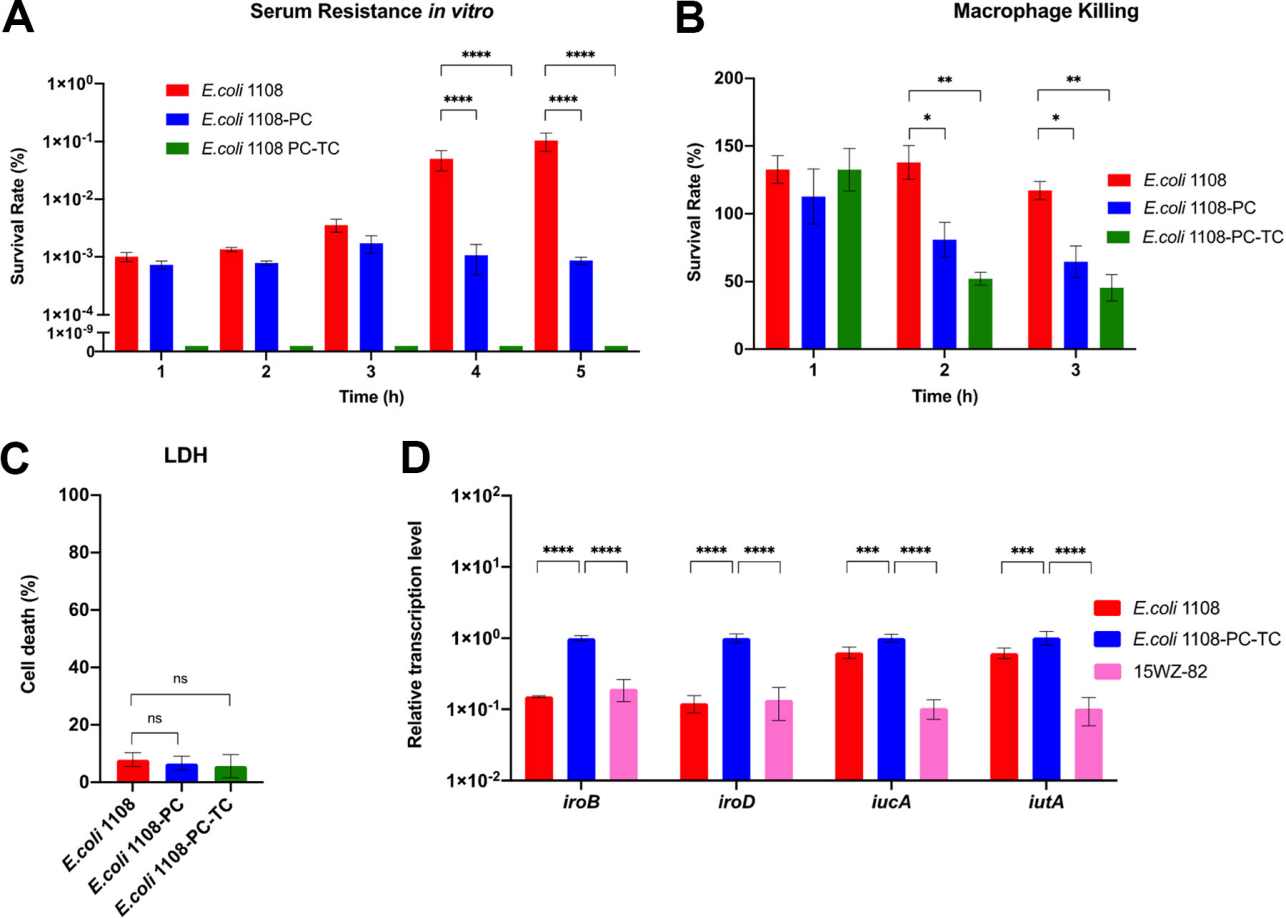
The virulence level of three test strains *in vitro*. The survival rate of different E. coli strains treated with serum *in vitro* (A) and RAW264.7 (cell/bacteria ratio of 1:5) (B). (C) The cell death rate of RAW264.7 infected with E. coli test strains. (D) The transcription levels of virulence genes in EC1108, EC1108-PC-TC, 15WZ-82. Tukey's multiple-comparison test was conducted. *, *P* < 0.05; **, *P* < 0.01; ****, *P* < 0.0001; ns, not significant.

To investigate the role of the virulence plasmids p1108-IncFIB and p15WZ-82_Vir in encoding virulence in E. coli, we tested three E. coli strains. EC1108-PC with the virulence plasmid p1108-IncFIB cured was used as a control. Strain EC1108 was considered the carrier of the virulence plasmid p1108-IncFIB in the EC1108-PC background, and EC1108-PC-TC was the EC1108-PC strain carrying a virulence plasmid from K. pneumoniae, p15WZ-82_Vir. Serum resistance assay was first conducted on these three strains, namely, EC1108, EC1108-PC-TC, and EC1108-PC, with results showing that the survival rate of EC1108 was higher than that of EC1108-PC and EC1108-PC-TC from hour 1 to 5, especially during hour 4 to 5, indicating that p1108-IncFIB confers high-level serum resistance *in vitro* in E. coli. However, EC1108-PC-TC was very sensitive to serum killing because almost no survival was detected within the 5 h assay period (Fig. 5A). The ability of the inherent virulence plasmid p1108-IncFIB in EC1108 to encode high-level resistance to serum might be attributed to the increased serum survival factor-encoding gene that it harbors (*iss*). The p15WZ-82 from Klebsiella pneumoniae could not cause an increase in serum resistance in EC1108-PC, but instead render this strain more sensitive, indicating that the virulence plasmid from K. pneumoniae, p15WZ-82_Vir, might impose a fitness cost in E. coli.

Next, a macrophage-killing assay was performed. Around 1 × 10^6^ CFU of the three E. coli strains were incubated with 2 × 10^5^ RAW264.7 cells for 3 h and the survival rate of the bacterial strains was recorded every hour. In the first hour, there was almost no difference in the survival rate of the three strains; at the end of the second hour, the survival rate of EC1108 was the highest, followed by EC1108-PC, and then EC1108-PC-TC. At the end of the 3rd hour, significant differences between the survival rate of the test strains were observed with EC1108 being the highest and EC1108-PC-TC being the lowest (Fig. 5B). After studying the phagocytosis of the three tested strains by macrophages, we also evaluated the effect of the three strains on the cytotoxicity of macrophages using LDH assay. It was found that after 3h infection of macrophages with the same number of bacteria, the death rate of macrophages caused by these three strains was very low and there was no significant difference between them suggesting that these E. coli strains did not cause a cytotoxic effect on macrophage cells (Fig. 5C). Taken together, it suggested that the different survival of these E. coli strains in RAW264.7 cells was indeed due to the resistance to macrophage killing.

Because it was found that EC1108-PC-TC seemed unable to acquire higher viability and virulence through serum and Raw264.7 resistance experiments, qRT-PCR was performed to further analyze the changes in the transcription level of virulence genes of p1108-IncFIB in EC1108 and p15WZ-82_vir in EC1108-PC-TC. As a control, the K. pneumoniae strain 15WZ-82 carrying p15WZ-82_vir was also tested. The genes *iroB*, *iroD*, *iucA*, and *iutA* shared by p1108-IncFIB and p15WZ-82_Vir were selected for further analysis. Compared with EC1108 and 15WZ-82, transcription levels of all these virulence genes were significantly upregulated in EC1108-PC-TC (Fig. 5D). These data suggested that the lower virulence of EC1108-PC-TC was not due to the inability to express key virulence genes encoding on virulence plasmid p15WZ-82_vir in E. coli. The inability of p15WZ-82_vir to mediate elevated virulence in E. coli may be due to the overexpression of these virulence genes leading to fitness cost in EC1108-PC-TC. The biosynthetic burden associated with plasmid carriage not only arises from gene transcription but may also be due to the low efficiency of gene translation or the inability of plasmid-encoded proteins to exert normal physiology in bacterial hosts, leading to the fitness cost imposed by p15WZ-82_vir in EC1108-PC-TC. (13) The exact mechanisms underlying the lower level of survival in serum and macrophage cells by E. coli carrying p15WZ-82_vir need further investigation.

### High virulence potential of EC1108 in a mouse model.

**FIG 6:**
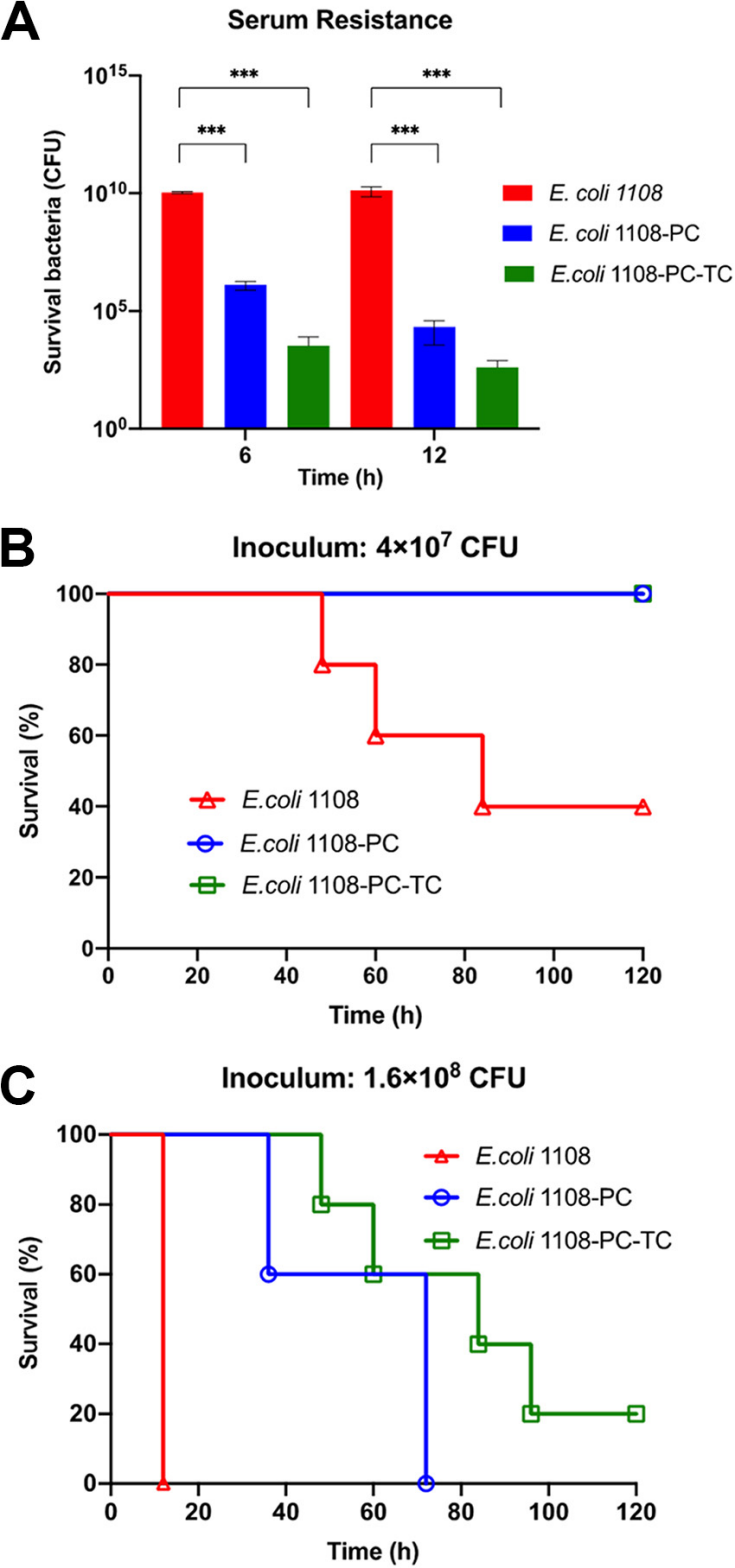
The virulence level of three test strains is depicted in a mouse model. (A) The number of bacteria that survived in mouse blood. Tukey's multiple-comparison test was conducted between EC1108 and the other two test strains. ***, *P* < 0.001; ****, *P* < 0.0001. Virulence potential of three strains in the mouse bacteremia infection model infected with 4 × 10^8^ (B) and 1.6 × 10^8^ CFU (C) of the test strains. The survival of mice infected by each test E. coli strain is depicted. The test strains included EC1108, EC1108-PC, and EC1108-PC-TC. A log-rank (Mantel-Cox) test conducted for the survival curves revealed significant differences (*P* < 0.001) between EC1108, EC1108-PC, and EC1108-PC-TC.

A mouse model was established to test the virulence potential of the three E. coli strains. First, the survival rate of E. coli strains in mouse blood was measured upon intravenous injection of 2 × 10^8^ CFU of different E. coli strains into five mice. At 6 h and 12 h, 500 μL of blood was withdrawn from the mice and the number of viable E. coli cells was determined. Our data showed that at 6 h, the CFU of EC1108 increased to around 10^10^, while those of EC1108-PC and EC1108-TC-PC dropped to 10^5^ and 10^3^, respectively. At 12 h, the CFU of EC1108 remained unchanged, whereas those of EC1108-PC and EC1108-TC-PC dropped further (Fig. 6A). In the mouse sepsis model, inoculation of 4 × 10^7^ CFU EC1108 resulted in 60% mortality at 84 h, whereas 0% mortality was recorded for EC1108-PC and EC1108-PC-TC. When injected with a higher dose (1.6 × 10^8^ CFU), a 100% mortality rate of EC1108 was recorded at 12 h, whereas the mortality of EC1108-PC and EC1108-PC-TC at 48 h was found to be 40% and 20%, respectively. At 120 h postinfection, all the mice in the EC1108 and EC1108-PC group were dead, whereas 20% of the mice in the EC1108-PC-TC group were still alive (Fig. 6B and C). These data were consistent with the results of *in vitro* serum resistance and macrophage resistance assays, suggesting that plasmid p1108-IncFIB confers high-level virulence in E. coli, whereas the acquisition of the K. pneumoniae*-*derived virulence plasmid p15WZ-82_Vir did not enhance the virulence and could also be associated with the fitness cost in E. coli.

## DISCUSSION

Avian pathogenic Escherichia coli (APEC) is an extraintestinal pathogenic Escherichia coli (ExPEC) strain that harbors a variety of plasmid-borne virulence factors. APEC is known to cause local and systemic infections in poultry, including multiple poultry colibacillosis, causing a reduction in meat and egg production and often poultry death (14, 15). It was reported that APEC inflicted huge economic losses on the poultry industry worldwide (16). In this study, we investigated the role of the virulence plasmid p1108-IncFIB in conferring virulence in the E. coli strain 1108, a highly virulent and resistant APEC strain, and found that p1108-IncFIB is indeed responsible for the expression of high virulence potential in E. coli both *in vitro* and *in vivo*. The virulence plasmid p1108-IncFIB harbors a variety of virulence factors, including genes encoding the iron/manganese ABC transporter (*sitABCD*), aerobactin (*iucABCD-iutA*), salmochelin (*iroBCDEN*), hemolysin (*hlyF*), and the increased serum survival factor (*iss*). These virulence genes are determinants of the high virulence potential of E. coli 1108. Iron is vital for the survival of E. coli, and the APEC strain has evolved a variety of strategies to sequester iron from the host. The ABC transport system encoded by *sitABCD* is involved in the process of iron uptake from free heme or heme-containing proteins. Salmochelin and aerobactin, encoded by *iroBCDEN* and *iucABCD* gene clusters, respectively, are two different types of siderophores involved in indirect iron acquisition (17–20). These transporters and siderophores mediate APEC adhesion, invasion, colonization, expression of other virulence genes, and persistence in the host (21). On the other hand, the serum survival factor (*iss*) can help evade the bactericidal effects of serum and cause sepsis (22–24). A high survival rate of bacteria in serum results in high mortality of the host. In addition, some APECs exhibit an increase in biosynthesis of outer membrane vesicles (OMV) through the acquisition of the *hlyF* gene. In the OMV biosynthesis process, the outer membrane protein is integrated into the OMV, where the proteins are scavenged from the cell membrane, causing a partial loss of the function of these cell membrane proteins (25). In this way, EC1108 may be able to evade the bactericidal effect of macrophages.

Due to the high degree of genetic similarity between APEC and human ExPEC, we speculate that E. coli strain 1108 may also cause human infections. Regarding their serotype and ST type, some studies found that more than 30% of APEC and human ExPEC strains were nondifferentiable (NT) at the somatic antigen (O). Also, ST359 was shared by both human ExPEC and APEC, for which PFGE patterns of >70% similarity were observed (26). A study showed that common virulence genes (*iss* and *iutA*) were found when 200 UPEC and APEC strains were compared genetically (27). Another study also illustrated that the virulence genes *iroN*, *iucD*, *sitD,* and *iss* were commonly found in APEC, UPEC, and NMEC isolates (9). On the other hand, similar human ExPEC-defining virulence markers (*hlyF*, *iroN*, *iutA*, *iss*) were found in 129 ExPEC strains isolated from meat and eggshells (28). Because most of the genes carried by EC1108 are also harbored by human ExPEC, we expect EC1108 has a high potential to cause infections in humans.

In this work, we conjugated p15WZ-82_Vir from K. pneumoniae that contained virulence factors similar to those of p1108-IncFIB into virulence plasmid-cured strain EC1108-PC. We found that p15WZ-82_Vir could be recombined with the resistance plasmid p1108-NDM in EC1108-PC to form the fusion plasmid p1108-NDM_Vir. However, this conjugative fusion plasmid could not confer high virulence to non-K. pneumoniae strains. Instead, it could suppress the virulence level of the recipient strain. It was reported that p15wz-82_vir could undergo homologous recombination with other plasmids such as resistance plasmids through partial integration events. For example, the recombination event of pGH44_43 and p15WZ-82_Vir recently reported by our laboratory was similar to the process of recombination of p15WZ-82_Vir and p1108-NDM observed in this study. We showed that the plasmid pGH44_43 actively inserted the region encoding Retron-type reverse RNA-directed DNA polymerase into p15WZ-82_Vir to generate the intermediate plasmid, pGH44TC_int. A crossover homologous recombination then occurred, resulting in the fusion of the two plasmids to form a 327,581bp conjugative fusion plasmid, pGH44TC_Vir. The above recombination processes are regarded as hallmark features of the evolution of p15WZ-82_Vir, which renders it highly efficient in disseminating the virulence genes that it contains to other bacterial strains (12).

In most conjugation events, plasmid transmission between different species of bacteria was relatively inefficient. For hypervirulent K. pneumoniae, horizontal transfer of virulence plasmids was limited to hvKP clones, which exhibit lineage specificity in plasmid distribution (29). In recent years, several documented cases of virulence plasmid transfer among different species of bacteria have been reported. Gili et al. (30) reported the horizontal transfer of the Salmonella enterica serovar infantis virulence and resistance plasmid pESI to Escherichia coli. The transfer of virulence plasmid from K. pneumoniae to E. coli 600 was also reported (3). In this study, we also found that a virulence plasmid from K. pneumoniae was able to be conjugated to E. coli and fused with its resistance plasmid to form a hvKP-MDR plasmid. However, the fusion plasmid imposes a fitness cost when entering the new bacterial host. Such fitness cost is even higher when the plasmid is acquired by strains of different bacterial species. The main burden related to plasmid carriage might be affecting the expression of plasmid-borne genes (13). Gene transcription is not regarded as a major cost affecting plasmid gene expression (31), and the major cost appears to arise from gene translation or subsequent interactions between cellular networks and proteins (32). It was reported that the translation efficiency of plasmid genes would be reduced when the compatibility between codon usage by foreign genes and the available tRNA pool in bacterial hosts was low (33). In addition, the unpredictability of the potential impact on bacterial physiology of the plasmid-encoded proteins to host bacteria may not be conducive to horizontal gene transfer (HGT) (13). It is worth noting that the reduction of fitness can be alleviated through compensatory evolution over time, so that the fusion plasmid which contains virulence and resistance genes identified in this work may pose a threat to animal and human health in the future. Hence, the transmission of the virulence plasmid from K. pneumoniae to non-K. pneumoniae strains should still be a major concern.

In conclusion, we identified a virulence plasmid known as p1108-IncFIB from a foodborne E. coli strain. This plasmid harbored a range of virulence factors that are highly similar to those located in the typical virulence plasmid harbored by clinical hypervirulent Klebsiella pneumoniae strains. We found that the virulence level encoded by the two types of plasmids were comparable and that p1108-IncFIB could be transmitted to and encode high-level virulence in E. coli strains of various genetic backgrounds, whereas the hypervirulence-encoding plasmid from Klebsiella pneumoniae may undergo fusion with resistance-encoding plasmids in E. coli. However, such fusion plasmids impose a fitness cost in such a way that the recipient strain carrying the fusion plasmid does not encode high-level virulence and is highly sensitive to the host immune response. The further evolution trends of these virulence-encoding plasmids need to be monitored.

## MATERIALS AND METHODS

### Bacterial strains.

E. coli strain 1108 was recovered from a chicken meat sample purchased from a supermarket in Shenzhen, China. The Klebsiella pneumoniae strain, 15WZ-82, was obtained from a clinical sample of an old female patient in a hospital in Zhejiang Province, China.

### Plasmid curing.

The plasmid curing experiment allows direct comparison between the phenotype of a virulence plasmid-bearing strain (E. coli 1108) and the virulence plasmid-cured strain (E. coli 1108-PC) (34). Briefly, a 200 μL overnight culture of E. coli strain 1108 was inoculated into 20 mL LB broth containing different concentrations of SDS (0.5, 1, 2, 3, 4, or 5% wt/vol) and incubated at 37°C for 48 h. PCR was performed to test the presence of the *iucC* gene located in the virulence plasmid p1108-IncFIB. S1-nuclease PFGE and XbaI PFGE (pulsed-field gel electrophoresis) analyses were conducted to confirm the curing of p1108-IncFIB.

### Conjugation assay.

Conjugation assays were performed using EC600-TC (E. coli strain), which contains the Klebsiella pneumoniae virulence plasmid (p15WZ-82_Vir) and was used as the donor, and EC1108-PC, which was strain EC1108 with p1108-IncFIB being cured and was used as the recipient strain. Both EC600-TC and EC1108-PC were grown to log phase (OD ≈ 0.6) in LB Broth at 37°C. The donor and recipient cultures were mixed in a 1:4 donor-to-recipient ratio, inoculated onto the 0.45 μm membrane placed on the surface of an LB agar plate, and incubated at 37°C overnight (35). All bacteria on the filter membrane were scraped and resuspended in 1 mL physiological saline. Upon serial dilution, the bacterial suspension was spread onto transconjugants-selective MacConkey plates containing 2 μg/mL cefotaxime and 2 μg/mL potassium tellurite (K_2_TeO_3_) because the MIC of cefotaxime in the donor strain and that of potassium tellurite in the recipient strain was both <2.

PCR was then performed to confirm the presence of *rmpA2*, which was used as a marker of p15WZ-82_Vir in transconjugants (11). An antimicrobial susceptibility test was carried out to distinguish between the donor, recipient, and transconjugants. In addition, S1-nuclease PFGE and XbaI PFGE were performed to further verify the identity of the virulence plasmid (p15WZ-82_Vir) recovered from the recipient strain (36). The diluted bacterial suspension mentioned above was spread onto a MacConkey plate containing 2 μg/mL cefotaxime to select the recipient strain. The total number of colonies of the recipient strain was recorded, and the conjugation efficiency was calculated by dividing the number of transconjugants against the number of recipient cells.

### Antimicrobial susceptibility test.

An antimicrobial susceptibility test was performed by using the broth microdilution method based on the guidelines of the Clinical and Laboratory Standards Institute (CLSI) (37). The antimicrobial agents tested in this study included amikacin, ampicillin, cefmetazole, cefotaxime, ciprofloxacin, cloxacillin, colistin, gentamicin, kanamycin, meropenem, potassium tellurite, and tetracycline. E. coli strain ATCC 25922 was considered the quality control strain. Two tests were conducted for each antimicrobial drug, and every test covered three biological replicates.

### DNA sequencing and bioinformatics analysis.

The whole genome of the test strains was extracted and sequenced through the Nanopore MinION platform (Nanopore, Oxford, United Kingdom) and the 150-bp paired-end Illumina NextSeq 500 platform (Illumina, San Diego, CA). Long and short reads were *de novo* hybrid assembled by Unicycler v.0.4.7 (38). Assembled genome sequences could be annotated with RAST v2.0 (39) and Prokka v1.12 (39). The insertion sequences and replicons were acquired from the Center for Genomic Epidemiology. The virulence and resistance genes were identified through the VirulenceFinder 2.0 and ResFinder4.1 services. EasyFig WIN_2.1 (40) and BLAST Ring Image Generator v.0.95.22 were used to compare plasmid similarity and plot plasmid maps, respectively.

### BLAST of similar plasmids and virulence genetic loci in E. coli genomes in GenBank.

To investigate the prevalence of three virulence gene clusters of the plasmid p1108-IncFIB among different sources in E. coli strains, we downloaded 2915 E. coli genome sequences which were mainly collected from 4 sources in China, including animal (poultry and swine), meat (pork chop and chicken), human clinical samples (blood, urine, sputum, and feces) and environmental samples deposited in the NCBI database (https://www.ncbi.nlm.nih.gov/pathogens). Draft genomes, including 1031, 637, 940, and 307 from animals, meat, clinical, and environmental samples, respectively, were used to screen virulence genes clusters *iroBCDEN*, *iucABCDiutA*, and *sitABCD* by BLAST (http://blast.ncbi.nlm.nih.gov/Blast).

### *In vitro* serum resistance assay.

A serum bactericidal assay was performed to test the ability of bacterial strains to resist serum killing. Bacterial strains were cultured in LB broth at 37°C and harvested in the early logarithmic phase (optical density [OD] approximately 0.3). Next, the bacterial suspension was adjusted to a concentration of 1 × 10^8^ bacteria/mL (OD approximately 0.18) of saline. A mixture of 50 μL of bacterial suspension and 150 μL of normal human serum was then incubated at 37°C in a 96-well plate (41). Viable bacterial counts were recorded at 0, 1, 2, 3, 4, and 5 h with duplications. Serum resistance curves were drawn by plotting the survival rate (log_10_ percentage) against time (h) using GraphPad Prism 8. A Tukey’s multiple-comparison test was conducted to analyze the difference between the survival rate of the three test strains.

### *In vitro* bacterial killing assay by macrophages.

Murine macrophage-like RAW264.7 cells were resuspended in DMEM and seeded at a concentration of 2 × 10^5^ cells per well on the 24-well plates. Cells were infected by the three types of test E. coli strains at a macrophage/bacteria ratio of 1:5 (Experiment group) and incubated at 37°C for 1, 2, or 3 h. DMEM medium containing the same number of bacteria (control group) was also incubated for the same period. 0.2% Triton X-100, a cell lysing reagent, was added to the mixture of the two groups. After serial dilution and overnight incubation on an LB agar plate, the number of bacteria was counted, and the bacterial survival rate was calculated by dividing the number of bacteria in the experiment group against the number of bacteria in the control group. The experiments were carried out at least twice, and each experiment included three biological replicates. A Tukey’s multiple-comparison test was carried out to analyze the difference between the survival rate of the three E. coli strains and the data were plotted by Prism 8.

### LDH cell death assay.

The cytotoxicity of macrophages infected with three test strains was evaluated by the lactate dehydrogenase (LDH) assay in culture supernatants. RAW264.7 cells were seeded on a 24-well plate at a density of 2 × 10^5^ cells per well and infected with three E. coli strains (MOI = 5) for 3 h. The supernatant was then collected and filtered through a 0.22 μm filter. The concentration of LDH released by macrophages was detected by spectrophotometry using the CyQUANT LDH Cytotoxicity assay kit (Invitrogen) following the manufacturer's instructions. The cell death rate can be calculated by the following formula: 100% × (experimental LDH activity−spontaneous LDH activity)/(maximum LDH activity−spontaneous LDH activity). Results were obtained from three experiments with three biological replicates in each group. Cell death rates were plotted by Prism 8, and the differences were analyzed by Tukey's multiple-comparison test.

### RNA extraction and quantitative PCR assay.

To assess the transcription of virulence genes in EC11108, EC1108-PC-TC, and 15WZ-82, overnight cultures were inoculated (1:100) into LB broth and grown at 37°C until the OD value reached 0.6. The total RNA of three strains was extracted by the RNeasy Minikit (Qiagen), and the Turbo DNA-free kit (Invitrogen) was used to remove DNA contaminants. Oneμg purified mRNA was reverse transcribed into cDNA by the SuperScript III First-Strand Synthesis SuperMix kit (Invitrogen), which was diluted at 1:20 for further qRT-PCR analysis. After that, qRT-PCR was carried out on an ABI QuantStudio 3 Flex real-time PCR system (Applied Biosystems) using the SYBR Green Supermix, and the primer pairs are shown in Table 3. Transcription levels of target genes were normalized to that of *gyrB* in Escherichia coli and Klebsiella pneumoniae, respectively. EC1108-PC-TC was used as a control for comparison. Gene transcription studies were performed three times, and each experiment included three biological replicates. The relative gene transcription levels were depicted by Prism 8, and a Tukey’s multiple-comparison test was conducted to analyze the difference between EC1108-PC-TC and the other two strains.

**TABLE 3:** Primers used in qRT-PCR

Primer	Sequence 5′ to 3′
iroB-F	ATCATCTACCCTCCGCTCG
iroB-R	ACGTCGATCCAGGCCAT
iroD-F	CATCTGCTTTCTGCCCCAC
iroD-R	CGCCACCATGCGTAATCGT
iucA-F	CGCATGGGTCGATGCTTA
iucA-R	ATAAACGCGCTGCCCTG
iutA-F	CGGTGGGTACACTCAGCTTC
iutA-R	CCCCTGCCTTTGTAGTCGT
gyrB-F (E. coli)	GACAAAGAACAGCACGGCAG
gyrB-R (E. coli)	CAAGCCACGCAGTTTCTCAC
gyrB-F (K. pneumoniae)	CGAACCGCTGGAAAAACTGG
gyrB-R (K. pneumoniae)	ATTCAGCTCGGAAACCAGGG

### Mouse bacteremia infection model.

A mouse infection experiment was performed to test the difference between the virulence level of the three E. coli strains (E. coli 1108, E. coli 1108-PC, and E. coli 1108-PC-TC) by determining the mortality rate in mice infected by each test strain. Six-week-old female ICR mice (weighing about 25 g) were purchased from the laboratory animal research unit of the City University of Hong Kong. The animals were randomly separated into several groups with five in each group. The immunosuppressive mice were obtained by subcutaneous injection of 150 mg/kg and 100 mg/kg cyclophosphamide in the first 3 days and 1 day of infection, respectively. The mice in different groups were intravenously injected with 4 × 10^7^ CFU and 1.6 × 10^8^ CFU of the corresponding strains, and their mortality rate in the next 120 h was recorded. Animal experiments were repeated twice to evaluate the consistency of the data. The survival curve was drawn by GraphPad Prism 8, and statistical comparison was conducted by the log‐rank (Mantel-Cox) test. All animal experiments were approved by the Laboratory Animal Research Unit of the City University of Hong Kong.

### Determination of bacteria load in mice blood.

Bacterial CFU in the blood of the test mice was recorded to further test the viability of these E. coli strains *in vivo.* Female ICR mice were divided into three groups (five mice in each group) and injected intravenously with 2 × 10^8^ CFU of the three test strains after cyclophosphamide treatment as previously described. Five hundred microliter of blood samples were collected after 6h and 12h, respectively, and spread onto an LB agar plate containing 20 μg/mL gentamicin upon serial dilution. Animal experiments were conducted twice to confirm the consistency of the data. A Tukey’s multiple-comparison test was carried out to analyze the difference between the bacterial concentration of donor strain, recipient strain, and transconjugants, and the results were presented by using Prism 8. All animal experiments were approved by the Laboratory Animal Research Unit City university of Hong Kong.

### Data availability.

The plasmid sequence data of p1108-NDM_Vir has been deposited in the GenBank under the accession number OL763373. All other data associated with this study are available upon request.
